# Correlation between Baseline 25(OH) Vitamin D Levels and Both Humoral Immunity and Breakthrough Infection Post-COVID-19 Vaccination

**DOI:** 10.3390/vaccines10122116

**Published:** 2022-12-10

**Authors:** Rami Abu Fanne, Ghalib Lidawi, Emad Maraga, Mahmud Moed, Ariel Roguin, Simcha-Ron Meisel

**Affiliations:** 1 Leumit Health Services, Tel Aviv 6473817, Israel; 2 Heart Institute, Hillel Yaffe Medical Center, Hadera 3810101, Israel; 3 Urology Department, Hillel Yaffe Medical Center, Hadera 3810101, Israel; 4 Clinical Biochemistry Department, Hadassah Medical Center, Jerusalem 9103102, Israel

**Keywords:** vitamin D, humoral response, breakthrough infection, vaccination

## Abstract

Objective: Vaccines against COVID-19 induce specific antibodies whose titer is perceived as a reliable correlate of protection. Vitamin D confers complex regulatory effects on the innate and adaptive immunity. In this study, we explored a plausible impact of baseline vitamin D content on achieved immunity following COVID-19 vaccination. Methods: A retrospective observational study comprising 73,254 naïve subjects insured by the Leumit Health Service HMO, who were vaccinated between 1 February 2020 and 30 January 2022, with one available vitamin D level prior to vaccination, was performed. The association between 25(OH) vitamin D levels, SARS-CoV-2 antibody titer, and post-vaccination PCR results were evaluated. Results: Of the study population, 5026 (6.9%) tested positive for COVID-19. The proportion of low 25(OH)D levels (<30 ng/mL) was significantly higher in the PCR-positive group (81.5% vs. 79%, *p* < 0.001). Multivariate analysis showed a higher incidence of breakthrough infection among non-smokers [1.37 (95% CI 1.22–1.54, *p* < 0.001)] and lower incidences among subjects with sufficient 25(OH)D levels (>30 ng/mL) [0.87 (95% CI 0.79–0.95, *p*—0.004)], hyperlipidemia [0.84 (95% CI 0.76–0.93, *p* < 0.001], depression [OR-0.87 (95% CI: 0.79–0.96, *p* < 0.005], socio-economic status >10 [0.67 (95% CI 0.61–0.73, *p* < 0.001)], and age >44 years. SARS-CoV-2 antibody titers were available in 3659 vaccinated individuals. The prevalence of antibody titers (<50 AU) among PCR-positive subjects was 42% compared to 28% among PCR-negative subjects (*p* < 0.001). Baseline 25(OH)D levels showed an inverse relation to total antibody titers. However, no association was found with an antibody titer <50 AU/mL fraction. Conclusion Baseline 25(OH)D levels correlated with the vaccination-associated protective COVID-19 immunity. Antibody titers <50 AU/mL were significantly linked to breakthrough infection but did not correlate with 25(OH)D levels.

## 1. Introduction

The dissemination of SARS-CoV-2 infections presented an unprecedented major health burden, which unrelentingly challenged the capacity of healthcare resources worldwide. The launch of the COVID-19 vaccines was a major step toward halting COVID-19 spread and limiting its mortality. COVID-19 vaccines elicit both humoral and cellular T-cell responses [1,2] but demonstrate a drop in effectiveness after six months [3,4,5,6,7]. These data contributed to the decision in the fall of 2021 to implement the BNT162b2 vaccine booster shot [8].

In the complex landscape of COVID-19, vaccine access inequities and limited revaccination compliance on one hand and the acceleration of herd immunization on the other, adjuvant approaches to hasten protective immunity could be helpful [9]. In this regard, several studies have pointed to the role of plasma 25(OH)D in COVID-19 immunization, since 25(OH)D deficiency has been associated with increased morbidity or mortality in COVID-19 patients [10,11]. Low 25(OH)D levels are common in the elderly, the obese, and among individuals with darkly pigmented skin [12]; indeed, these populations proved to be more vulnerable to COVID-19 infection with disproportionately high morbidity and mortality [13,14].

In this study, we describe the results of a large population-based data analysis evaluating the impact of baseline plasma 25(OH)D content on vaccine-related antibody response and breakthrough infection.

## 2. Methods and Patients

We conducted a population-based study among adult members of the Leumit Health Services (LHS), a large, Israeli nation-wide health maintenance organization (HMO), which provides health services to nearly 730,000 members. LHS has a comprehensive, computerized database, which is continuously updated regarding the demographics, medical diagnoses and encounters, hospitalizations, and laboratory tests of insured members.

The socio-economic status (SES) was defined according to the home address. The Israeli Central Bureau of Statistics classifies all cities and settlements into 20 levels of SES. The one to nine classifications are considered low to medium SES, while the ten to twenty higher classifications are considered medium to high SES. Ethnicity was also defined according to the home address of the HMO members and categorized into three groups: general population, ultra-orthodox Jews, and Arabs.

All LHS members have identical health insurance coverage and access to healthcare services. Relevant diagnosis is entered or updated according to the International Classification of Diseases 10th revision (ICD-10). The validity of chronic diagnoses in the registry has been previously established (Hamood et al., 2016; Rennert and Peterburg, 2001). The study population included all LHS members aged 18 or older who fulfilled the following criteria:

(1) Received two vaccine injections (without documented prior infection) between the first of February 2020 and the 30th of January 2022.

(2) Were tested for plasma 25(OH)D level at least once prior to vaccination. The median duration (IQR) between 25(OH)D assay and SARS-CoV-2 positivity was 5 (3–7) months. Notably, patient supplementation history was not readily available and was not accounted for as part of our research.

(3) Underwent RT-PCR testing at least two weeks after the second vaccination and before the next booster, if any.

We extracted available SARS-CoV-2 serology and associated demographic and clinical data for all study subjects. SARS-CoV-2 RT-PCR testing followed the Israeli Ministry of Health instructions to perform COVID-19 testing upon physician referral based on clinical criteria of exposure to confirmed COVID-19 patients or in the presence of symptoms suggestive of COVID-19 infection. The Allplex 2019-nCoV assay (Seegene, Seoul, Republic of Korea) was used until March 10, 2020, followed by the COBAS SARS-CoV-2 6800/8800 assay (Roche Pharmaceuticals, Basel, Switzerland). Referrals to SARS-CoV-2 antibody testing against spike proteins were left to the discretion of the treating physician. The test results were not intended to determine the need for vaccination. The Abbot Alinity™ i system (Illinois, IL, USA) was employed for antibodies testing. Antibody levels measured by this test below 50 AU/mL were considered non-protective. In internal testing, the Abbott Alinity™ system showed reliable results, with 99.6% specificity and 100% sensitivity for COVID-19 patients tested 14 days after the initial symptoms [15]. The Abbott assay was validated externally [16] with excellent sensitivity and specificity. Qualitative results and index values reported by the system were used in the analyses.

Baseline medical conditions known to be associated with the severity of COVID-19 infection or the antibody level in the adult population, including obesity, diabetes mellitus, hypertension, asthma, chronic obstructive pulmonary disease, ischemic heart disease, presence of malignancy, and chronic kidney disease, were extracted. Obesity was defined as having a BMI > 30 m^2^/kg. As for the categorization of vitamin D levels, we adopted in the present study the common conventions of the American Endocrine Society, National Osteoporosis Foundation, and International Osteoporosis Foundation [17,18]. In this scheme, values lower than 20 ng/mL represented vitamin D deficiency, concentrations of 21–29 ng/mL were considered insufficient, and values >30 ng/mL reflected adequate levels. Study protocol was approved by the LHS Institutional Review Board (13-21-LEU).

## 3. Statistical Analysis

Descriptive statistics in terms of mean, standard deviation, median, and percentiles were presented for all parameters in the study. Differences between groups (positive PCR vs. negative PCR, antibody titer <50 vs. antibody titer >=50) were presented by *t*-test or Fisher exact tests for continuous and categorical parameters, respectively. Differences within groups (positive PCR or negative PCR) according to vitamin D levels and time interval from second vaccination were calculated with Pearson Chi-square. As the distributions of the antibody levels were not normally distributed (by Kolmogorov–Smirnov test), we used the Log-transformed function. Multi-level assessments of vitamin D, PCR status, antibody levels, and the time interval elapsed from vaccination were calculated using Kruskal–Wallis tests with multiple comparisons. Parameters were selected as candidates for the multivariate analysis on the basis of their significance from the univariate analysis. The multivariate logistic regression model was assessed to determine the effect of the independent parameters associated with positive PCR. *p* < 0.05 was considered significant. IBM^®^SPSS version 28 was used for all statistical analyses.

## 4. Results

**Figure 1:**
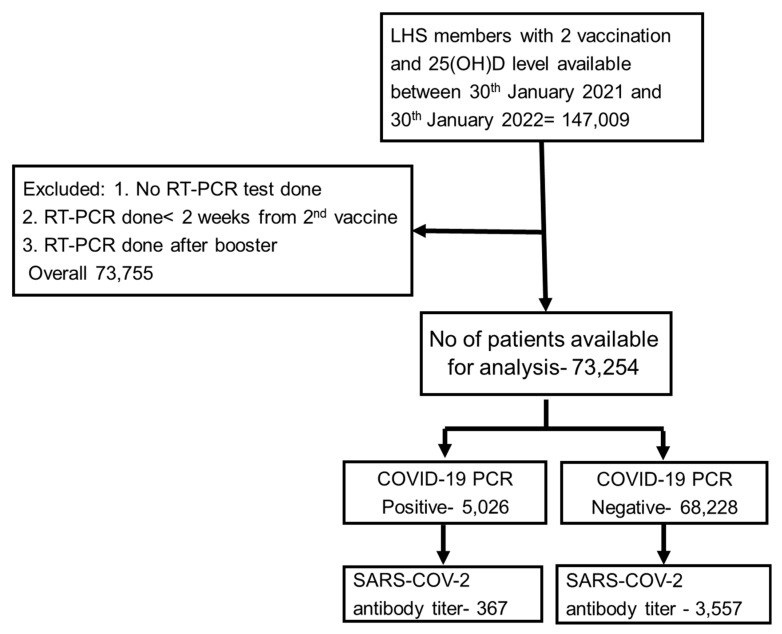
Scheme of the study flowchart.

During the study period, 147,009 fully vaccinated adults with no documented evidence of previous COVID-19 infection, who had available baseline plasma 25(OH)D levels, were identified. Of the fully vaccinated group, 73,254 adults who fulfilled the inclusion criteria comprised the study cohort. Figure 1 displays the flow diagram used for cohort selection, while their characteristics at inclusion are shown in Table 1.

Among the study group, the breakthrough infection rate at a median follow-up of 6.2 months (IQR: 5.4–6.8 months) was 6.9%. The cohort characteristics and comorbidities stratified by their PCR status are shown in Table 1.

**Figure 2:**
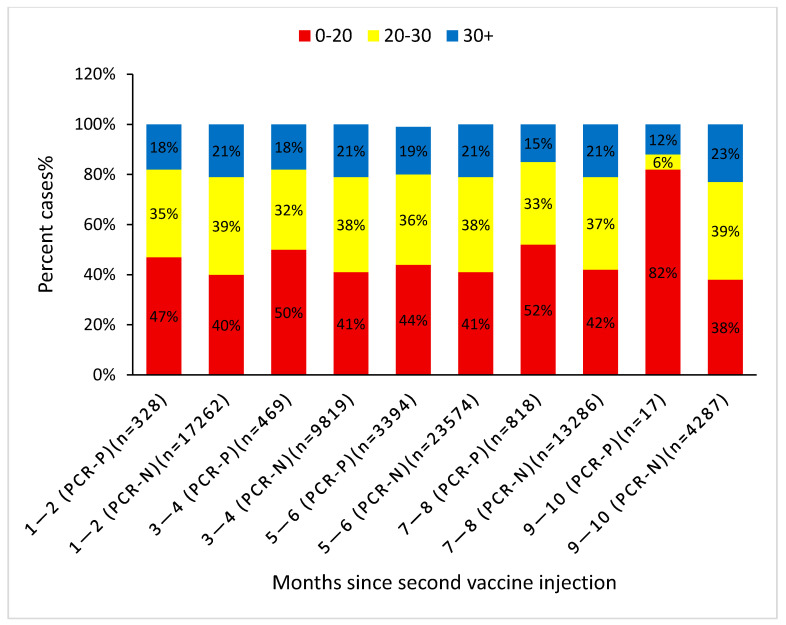
Percent cases with negative/positive RT-PCR classified by baseline 25(OH)D levels at each time point following the second vaccination. The Pearson Chi-square test was applied here.

Primary univariate analysis proved younger age, female gender, Arab and Orthodox Jewish ethnicity, asthma, low SES, and non-smoking were significantly associated with breakthrough infections. Interestingly, comorbidities including depression, chronic renal failure, CVA, PVD, IHD, hyperlipidemia, diabetes, and COPD were negatively linked to breakthrough infections (*p* < 0.05). The proportion of subjects with low baseline 25(OH) vitamin D level (<30 ng/mL) was significantly higher among subjects who developed breakthrough infections (81.5% vs. 79%, *p* < 0.001). Figure 2 includes patient numbers and shows the association between baseline vitamin D level categories with PCR status (positive or negative) at different periods following vaccination. At each period after vaccination, the proportion of low 25(OH)D subjects is higher among the PCR-positive subjects representing the breakthrough infection group. At 9–10 months, the positive PCR results accounted for only 0.3% of the total positive PCR results, which is why the relative impact of this time period on the total result was trivial. The infectivity rate at each specific time period with each vitamin D level category is shown in Table 2. At each time interval (until months 9–10), the lower the vitamin D level, the higher the infectivity rate. The overall infectivity rate at vitamin D levels <20, <30, and >30 ng/mL was 7.8%, 7.1%, and 6%, respectively, with *p* < 0.05. The infectivity rate in the face of insufficient vitamin D levels (20–30 ng/mL) was marginally higher than that in the sufficient vitamin D group (6.3% vs. 6.1% (*p* = NS)).

A multivariate regression model applied after adjusting for demographic variables and comorbidities (all the characteristics that were found significant in Table 1) showed a significant negative correlation between sufficient baseline serum 25(OH)D levels (>30 ng/mL) and breakthrough infections [OR 0.87 (95% CI: 0.79–0.95, *p* = 0.004)]. A significant negative correlation was also found between baseline 25(OH)D levels >20 ng/mL and breakthrough infections [OR 0.89 (95% CI: 0.784–0.985, *p* = 0.027)]. The independent negative correlates of breakthrough infections were hyperlipidemia [OR 0.84 (95% CI: 0.76–0.93, *p* < 0.001], depression [OR 0.87 (95% CI: 0.79–0.96, *p* < 0.005], medium-high SES (>10) [OR 0.67 (95% CI: 0.61–0.73, *p* < 0.001)], and age older than 44 years. The risk of COVID-19 breakthrough infections was independently positively associated with the non-smoking category [OR 1.37 (95% CI: 1.22–1.54, *p* < 0.001)].

We then evaluated the impact of SARS-CoV-2 antibody values drawn after the second vaccination and before any booster injection on the RT-PCR results and its relation to baseline 25(OH)D levels. Of the 3659 subjects with available antibody results, 340 were associated with positive RT-PCR and 3319 with negative ones.

**Figure 3:**
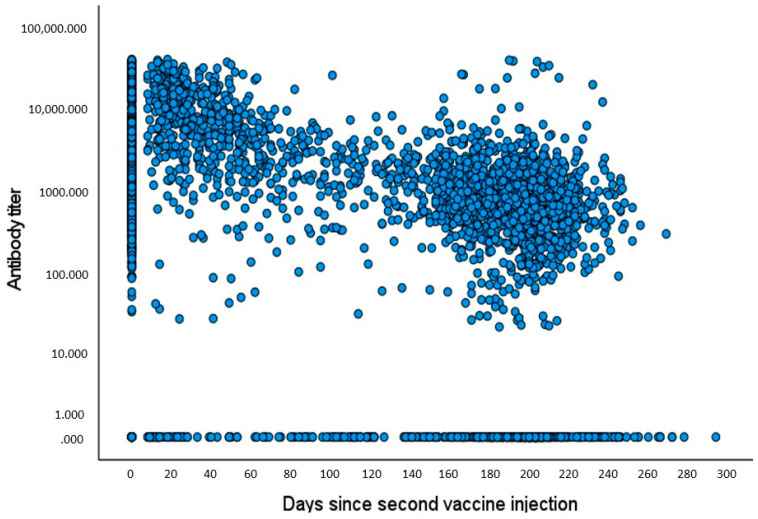
Scatter plot (BIVAR): antibody titers plotted against elapsed time since 2nd vaccination. We used the Pearson correlation with the logarithmic scale to test the relation between antibody titer and days since second vaccine injection.

**Figure 4:**
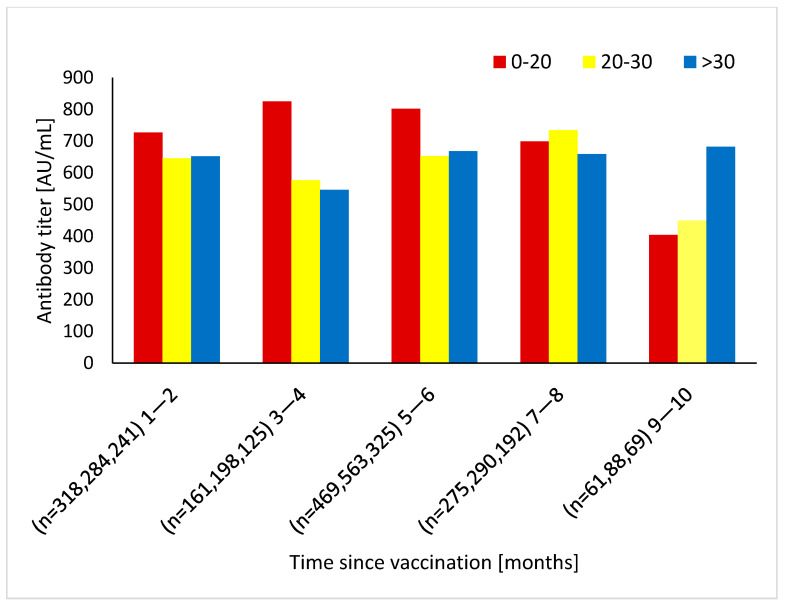
Antibody titers from vaccinated groups stratified by baseline 25(OH)D levels at each time point since 2nd vaccination. The Kruskal–Wallis tests with multiple comparisons was used to compare between the groups.

The evolution of antibody levels as a function of the time that elapsed since vaccination showed a gradual and continuous decline of antibody levels, approaching 39% of the baseline level after 9 months (Figure 3). The temporal decrease in antibody levels over time was also previously shown. In fact, a different research group used the present large-scale population and demonstrated a similar scatter plot with values showing a quick drop in titers, approximately decreasing by 40% with each passing month [4]. Since the pattern of titer decay has already been evaluated in the current population, we focused on the relation between vitamin D levels and antibody titers at successive periods following the vaccination (Figure 4). We observed a non-significant tendency toward higher titers during the first 6 months post-vaccination associated with deficient vitamin D levels (0–20 ng/mL); then, we observed a slow antibody titer decline until 8 months, followed by a pronounced decay (*p* = NS). In the subject group characterized by sufficient 25(OH)D levels, the antibody titer was preserved throughout the study period (*p* = 0.24). However, this pattern was not reflected in Figure 3 (the scatter plot), since the high baseline 25(OH)D group (>30 ng/mL) comprised only a minority of study patients (about 25%).

**Figure 5:**
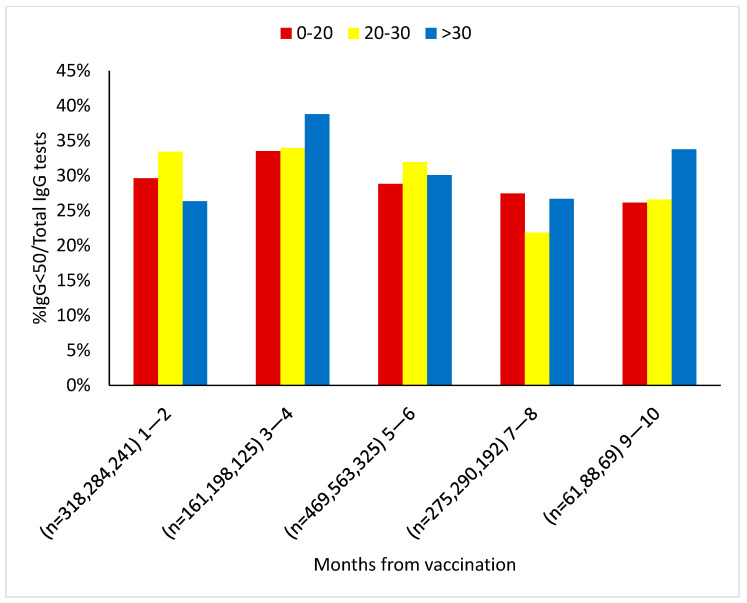
% Antibody titer <50 AU/mL from the vaccinated group stratified by baseline 25(OH)D levels at each time point since the 2nd vaccination. The Pearson Chi-square test was applied here.

Further testing for a possible association between the antibody threshold <50 AU/mL and plasma 25(OH)D levels did not yield a consistent correlation with baseline plasma 25(OH)D levels (Figure 5). However, it did show, as described above, reasonably stable antibody values in the sufficient vitamin D subset. Nevertheless, we found a positive relationship between the prevalence of antibody titers <50 AU/mL and the incidence of breakthrough infections, which was 42% among the positive RT-PCR subjects (154 out of 367 PCR positive tests) and 28% among the negative RT-PCR subjects (1013 out of 3557 PCR negative tests) (*p* < 0.05).

In the absence of a significant association between total antibody titer and the occurrence of breakthrough infections, we conducted a multivariate analysis that included the dependent variable of antibody titer (< or >50 AU/mL). Table 3 depicts the correlation between different independent baseline characteristics and antibody titers < or >50 AU/mL. Significant predictors of antibody titer <50 AU/mL included gender, ethnicity, nephrotic syndrome, CRF, and low SES. As observed in the case of total antibody titer, this antibody threshold was unaffected by the plasma 25(OH)D (*p* = 0.87). Further mapping of the percentage of antibody titers <50 by baseline concentration of 25(OH)D, showed that higher percentages coincided with higher contents of 25(OH)D.

### Timing of COVID-19 Infection and the Effect of Baseline Vitamin D Level

Of the RT-PCR-positive study patients, a large surge in infections was noticed among study patients at 5 to 6 months post-vaccination (67.6% of total documented infections).

The prevalence of low 25(OH)D levels (<30 ng/mL) among the infected subjects was 81.5%, compared to 78.9% among the negative RT-PCR cases (*p* < 0.001).

## 5. Discussion

Both the BNT162b2 mRNA vaccine and the COVID-19 infection trigger a robust immune response in most people. However, numerous studies have recently documented a “rapid decay” in antibody titer within a few months [3,4,5,6,7]. The latter observation raised the issue of the durability of vaccine-induced immunity to the coronavirus. In this context, vitamin D was suggested as a novel adjuvant to augment COVID-19 immunogenicity.

Recently, several trials have investigated the approach of improving COVID-19 outcomes through vitamin D supplementation, yielding controversial results. Vitamin D substitution reduced cough duration [19], shortened hospital stay, and decreased mortality in COVID-19 patients [20]. In contrast, a single high dose of Vitamin D3 in hospitalized moderate to severe COVID-19 patients showed a null effect [21]. The effect of vitamin D on adaptive humoral immunity is unclear. Chen and colleagues [22] found an inverse relationship between vitamin D levels and the measles antibody titer. A German group tested 126 healthy adult volunteers with moderate vitamin D levels and reported that SARS-CoV-2 antibody concentration did not depend on 25(OH)D content [23]. On the other hand, Anastasia et al. [24] tested 712 subjects for SARS-CoV-2 antibodies three months after the second dose of the BNT162b2 vaccine and found a positive association between total antibody titer and baseline vitamin D levels.

The protective role of vitamin D in the primary prevention of COVID-19 infection has previously been reported [25]. We used a data set from the same integrated healthcare organization, involving more than 73,000 fully vaccinated individuals, to evaluate the potential impact of baseline 25(OH)D contents on immunogenicity-related endpoints post-vaccination. A valuable finding of the current study is the relevance of 25(OH)D as a factor associated with the secondary prevention of post-vaccination breakthrough infection.

In line with a former study from LHS [25] completed before the launch of the COVID-19 vaccination, we found that older age, smoking, and high SES inversely correlated with breakthrough infections after vaccination. Older age was associated with higher adherence to COVID-19 preventive measures, including social distancing [26]. This behavior may explain the low infectivity rate in older age people. Surprisingly, although smoking is an established risk factor for contracting lung infections [27,28] and the fact that smokers were negatively impacted when contracting SARS-CoV-2 infection [29], consolidated epidemiological data has reported a low prevalence of active smokers among COVID-19 patients, including at the population level [30,31,32,33,34]. On one hand, a protective effect of nicotine interaction with the immune and renin–angiotensin systems was suggested [35]. On the other, several methodological biases might have influenced this negative association, including underrepresented smoking prevalence [29,36], un-adjustment of potential confounding factors (comorbidities, age, gender, SES, and occupation) [36], and strict adherence to protective measures against COVID-19 among smokers [33]. Furthermore, smokers were shown to more likely self-report COVID-19 symptoms, leading to higher volumes of RT-PCR tests than non-smokers [34].

A strong association between SES and COVID-19 incidence has already been pointed out [37,38]. The critical consequences of socio-economic inequalities on COVID-19 outcomes are believed to result from the combination of overcrowded accommodations (an established risk factor for lower respiratory tract infections [39] and the major limiting factor of physical/social distancing), employment in occupations that do not provide opportunities to work from home, and financial uncertainty-related stresses that make the immune system more prone to sustain infections [40,41]. Notably, multivariant analysis proved both IHD and COPD to be associated non-significantly with testing positive for SARS-CoV-2 infection.

Although it was included by the centers for disease control and prevention as one of the conditions that confers a higher risk for severe COVID-19 infection [42], we found that depressive subjects were less likely to become infected with the virus. This observation might be explained by the combination of both a high rate of smoking [43] (already shown to be inversely linked with infection) and a higher adherence to precautionary measures, including wearing face masks, frequent handwashing, household disinfection, social distancing, minimizing unnecessary travel, and stocking up on food and daily essentials among this population [44]. Furthermore, reduced risk of breakthrough infection was substantially linked to hyperlipidemia. Statins are the most prescribed lipid-lowering agents for hypercholesterolemic individuals. Beneficial effects of statins on COVID-19-related outcomes were previously confirmed [45,46]. We believe that routine statin use in this group might have mitigated SARS-CoV-2 transmission. Regarding possible vitamin D associations with antibody kinetics, several observations should be stressed:

A total of 71% of subjects in the vaccinated group who had antibody tests <50 AU/mL had low vitamin D levels (<30 ng/mL) (Figure 5).

We demonstrated that vaccination induced a substantial anti-spike protein antibody response, with declining titers observed after an initial peak, although the magnitudes of the peak and decline were relatively inversely related to vitamin D levels (i.e., higher initial peak with deficient vitamin D levels and higher long-term titer with sufficient vitamin D levels).

In this context, several studies reported an inverse relationship between the serum level of 25(OH)D and virus antibody titer [22,47,48]. Mechanistically, vitamin D has been shown to hamper the production of immunoglobulins [49,50,51,52], attenuating the immune reaction induced by viruses. Such evidence suggests that insufficient vitamin D levels may lead to a more florid, though short-lived, antibody response. Overall, no significant consistent association was found between baseline 25(OH)D values and either anti-spike protein antibody total titer or anti-spike protein antibody more or less than 50 AU/mL.

Another key observation was the peak breakthrough infection rate at 5–6 months post-vaccination, confirming virus ability to escape vaccine-acquired immunity. Intriguingly, at this stage both the magnitude of antibody titers and the proportion of sufficient plasma 25(OH)D tests were similar to their levels at the immediate preceding months. This observation proves the complexity of the immunity puzzle: vitamin D and antibodies are simply just two measurable pieces of this puzzle. The human immune system has two levels of immunity: a non-specific, rapidly recruited innate immunity and the adaptive immune system, including antibodies, B cells, and T cells that launch a targeted pathogen-specific reaction.

Vitamin D has claimed pivotal immunological effects, including innate and adaptive immune system modulation [53] and regulation of the renin–angiotensin–aldosterone system [54,55]. In the innate immune arm, vitamin D upregulates the expression of the first line of defense against infectious agents: the antimicrobial peptide defensins and cathelicidins [56,57]. These pleiotropic effects mediate vitamin D’s role in the prevention of COVID-19 [58,59]. Several lines of evidence have suggested a central role of T cells in SARS-CoV-2 protective immunity; peripheral lymphopenia is an established surrogate indicator of a poor prognosis during COVID-19 infection [60]. Likewise, T cells were proved to mount a strong response to the virus’s “spike” protein, which is essential for host cell invasion [61]. Interestingly, the robust memory T cell responses were detectable, even in antibody-seronegative members [62].

The BNT162b2 mRNA vaccine relies solely on spike antigens, generating exclusively spike-specific memory. The peak in breakthrough infection encountered at 5–6 months post-vaccination occurred despite there being no observed difference in vitamin D categories and median antibody levels at the time intervals before and after this surge. This observation largely challenges the independent roles of the anti-spike protein antibody titer and vitamin D in predicting SARS-CoV-2 immunogenicity. A booster vaccine should be considered at this stage.

## 6. Study Limitation

Vitamin D levels are mostly tested as part of a routine blood workup, and in a minority of patients, it is undertaken following a clinical suspicion for vitamin D deficiency. Patient vitamin D supplementation history was not available as part of our research. Moreover, in April 2020, the Health Ministry published updated nutritional guidelines for the entire population, recommending the consumption of a daily supplement containing 800–1000 international units of vitamin D. As vitamin D supplement is one of the most common over-the-counter (OTC) purchases, the retrospective study design could not assess adherence to this recommendation. Of note, in the USA trial [63], supplementation of 1000 IU of vitamin D plus 2000 mg of calcium or placebo for 9 weeks had the same marginal effect on mean 25(OH)D levels. Neither group achieved sufficient concentrations. Considering seasonal fluctuations, a previous study from Israel [64] found low vitamin D to be common across all ages, genders, and seasons. Nevertheless, previous recent Israeli vitamin D results [25] and our cohort substantial association between prior vitamin D deficiency and COVID-19 disease outcome suggest that most vaccinated individuals who tested positive for a SARS-CoV-2 infection had low 25(OH)D values when contracting COVID-19. Furthermore, the time between vitamin D sampling and vaccination did not serve as a parameter in the statistical analysis. However, this is a typical limitation in studies performed in very large patient datasets, where minute individual details, occasionally important, are not available. The rationale behind these large-scale studies is that due to the large number of patients, individual variations tend to mutually cancel each other.

## 7. Conclusions

The immune response to COVID-19 is multifaceted, and reliable correlates of protection against COVID-19 are necessary. Currently, a serological response is perceived as the test of choice to assess protective immunity against COVID-19. Nevertheless, neither the type of antibodies nor the protective threshold is specified, and the present study challenges the presumed protective role of the anti-spike protein antibodies measured. Evolving COVID-19 variants are endowed by higher infectivity and greater capacity to evade antibody protection, limiting the durability of the current vaccine to prevent SARS-CoV-2 infection. The non-proportional surge in breakthrough infection rate at 5–6 months post-vaccination probably highlights the key importance of a multifaceted immune response, including sufficient vitamin D levels, various antibodies against multiple epitopes, and T cell immunity directed against conserved epitopes, in limiting SARS-CoV-2 infectivity, disease severity, and duration.

## Figures and Tables

**Table 1:** Baseline characteristics of the fully vaccinated group.

	PCR Status		
	Positive (*n* = 5026)	Negative (*n* = 68,228)	*p*-Value
Age	45.4 ± 17.8	50.8 ± 18.9	*p* < 0.001
Gender Male Female	(33%) (67%)	(37%) (63%)	*p* < 0.001
Ethnicity Arab Orthodox Jewish Other	(20%) (19%) (61%)	(13%) (16%) (71%)	*p* < 0.001
25(OH)D level 0–20 ng/mL 20–30 ng/mL >30 ng/ml	(46.5%) (35%) (18.5%)	(41%) (38%) (21%)	*p* < 0.001
Anxiety	(36%)	(36%)	*p* = 0.96
Schizophrenia	(2.0%)	(2.2%)	*p* = 0.52
Depression	(23.0%)	(26.1%)	*p* < 0.001
Dementia	(4.0%)	(4.4%)	*p* = 0.31
Nephrotic syndrome	(0.6%)	(0.4%)	*p* = 0.084
Chronic Renal failure	(6.5%)	(7.4%)	*p* = 0.072
CVA	(4.4%)	(6.0%)	*p* < 0.001
CHF	(4.8%)	(5.1%)	*p* = 0.46
PVD	(3.9%)	(5.3%)	*p* < 0.001
IHD	(9.0%)	(10.9%)	*p* < 0.001
Hyperlipidemia	(53.6%)	(63.4%)	*p* < 0.001
HTN	(2.0%)	(2.1%)	*p* = 0.88
DM	(11.7%)	(13.1%)	*p* = 0.032
COPD	(7.8%)	(10.5%)	*p* < 0.001
Asthma	(16.6%)	(14.9%)	*p* = 0.016
SES 1–10 10–20	(55%) (45%)	(43%) (57%)	*p* < 0.001
Smoking status Active smokers Non-smokers Former smokers	(14%) (84%) (2%)	(19%) (79%) (2%)	*p* < 0.0001
BMI 16.5–18.5 18.5–24.9 25–29.9 30+	(3%) (34%) (34%) (29%)	(3%) (34%) (35%) (28%)	*p* = 0.40

CVA, cerebrovascular accident; CHF, congestive heart failure; PVD, peripheral vascular disease; IHD, ischemic heart disease; HTN, hypertension; DM, diabetes mellitus; COPD, chronic obstructive pulmonary disease; SES, socio-economic state; BMI, body mass index.

**Table 2:** The infectivity rate at each specific time period with each specific vitamin D level. Significant differences are marked by an asterisk or hash mark (*: *p* < 0.05 [25(OH)D] < 20 vs. [25(OH)D] > 30; #: *p* < 0.05 [25(OH)D] < 30 vs. [25(OH)D] > 30).

	[25(OH)D] < 20	[25(OH)D] 20–30	[25(OH)D] > 30
1–2 Months	2.18% (*n* = 7067)	1.7% (*n* = 6826)	1.57% (*n* = 3694)
3–4 Months	5.6% (*n* = 4214)	3.87 (*n* = 3898)	3.77% (*n* = 2174)
5–6 Months	13.56% (*n* = 11,094)	11.94% (*n* = 10,291)	11.85% (*n* = 5579)
7–8 Months	7.13% (*n* = 5992)	5.1% (*n* = 5236)	4.35% (*n* = 2874)
9–10 Months	0.86% (*n* = 1637)	0.06% (*n* = 1672)	0.20% (*n* = 994)
1–10 Months	7.78% (*n* = 30,004) *	6.31% (*n* = 27,923) #	6.06% (*n* = 15,315)

**Table 3:** Baseline characteristics of the SARS-CoV-2 antibody tests of available, vaccinated participants according to < or >50 AU/mL values. The 3rd column includes the total number of antibody tests available.

	Antibody Titer < 50 (*n* = 1014)	Antibody Titer >= 50 (*n* = 2645)	Total; *n* = 3659	*p*
Age	51.8 ± 16.9	51.2 ± 16.5	51.4 ± 16.6	*p* = 0.32
Gender Code1 = male Code2 = female	(36%) (64%)	(31%) (69%)	(32%) (68%)	*p* = 0.005
PCR positive	13.2%	7.7%	9.3%	*p* < 0.05
Ethnicity Arab Orthodox Jewish Other	(13.5%) (7%) (79%)	(19%) (12%) (69%)	(17.5%) (10.5%) (72%)	*p* < 0.001
25(OH)D levels 0–20 ng/mL 20–30 ng/mL >30 ng/ml	(34%) (40%) (26%)	(34%) (41%) (25%)	(34%) (41%) (25%)	*p* = 0.87
Anxiety	(36%)	(40%)	(39%)	*p* = 0.11
Schizophrenia	(1.5%)	(1.3%)	(1.3%)	*p* = 0.71
Depression	(26.4%)	(26.5%)	(26.5%)	*p* = 0.96
Dementia	(1.9%)	(1.4%)	(1.6%)	*p* = 0.38
Nephrotic syndrome	(1.2%)	(0.3%)	(0.6%)	*p* = 0.016
Chronic Renal failure	(10.3%)	(5.8%)	(7.1%)	*p* < 0.001
CVA	(5.0%)	(3.7%)	(4.1%)	*p* = 0.13
CHF	(5.3%)	(4.6%)	(4.8%)	*p* = 0.48
PVD	(4.7%)	(4.2%)	(4.3%)	*p* = 0.52
IHD	(11.2%)	(9.8%)	(10.2%)	*p* = 0.28
Hyperlipidemia	(65.0%)	(63.1%)	(63.6%)	*p* = 0.37
HTN	(2.7%)	(2.0%)	(2.2%)	*p* = 0.30
DM	(11.0%)	(10.8%)	(10.8%)	*p* = 0.89
COPD	(10.7%)	(10.5%)	(10.5%)	*p* = 0.88
asthma	(14.9%)	(15.8%)	(15.5%)	*p* = 0.59
SES 1–10 10–20	(30.4%) (69.6%)	(40.8%) (59.2%)	(37.9%) (62.1%)	*p* < 0.001
Smoking status Active smoker Non-smoker Former smoker	(14.6%) (82.9%) (2.5%)	(14.0%) (84.3%) (1.7%)	(14.1%) (83.9%) (1.9%)	*p* = 0.27
BMI 16.5–18.5 18.5–24.9 25–29.9 30+	(2.6%) (36.7%) (36.4%) (24.3%)	(2.4%) (35.9%) (35.7%) (26.0%)	(2.5%) (36.1%) (35.9%) (25.5%)	*p* = 0.77

CVA, cerebrovascular accident; CHF, congestive heart failure; PVD, peripheral vascular disease; IHD, ischemic heart disease; HTN, hypertension; DM, diabetes mellitus; COPD, chronic obstructive pulmonary disease; SES, socio-economic state; BMI, body mass index.

## Data Availability

This study is based on real-world patient data, including demographics and comorbidity factors, that cannot be communicated due to patient privacy concerns.
